# Effect of Underlying Renal Disease on Nutritional and Metabolic Profile of Older Adults with Reduced Renal Function

**DOI:** 10.3389/fnut.2017.00004

**Published:** 2017-03-17

**Authors:** Silvia Lai, Maria Ida Amabile, Silvia Altieri, Daniela Mastroluca, Carlo Lai, Paola Aceto, Massimiliano Crudo, Anna Rita D’Angelo, Maurizio Muscaritoli, Alessio Molfino

**Affiliations:** ^1^Department of Clinical Medicine, Sapienza University of Rome, Rome, Italy; ^2^Department of Clinical and Molecular Medicine, Sapienza University of Rome, UOC Nephrology, Sant’ Andrea Hospital, Rome, Italy; ^3^Nephrology and Dialysis Unit, Hospital ICOT Latina, Sapienza University of Rome, Rome, Italy; ^4^Department of Dynamic and Clinic Psychology, Sapienza University of Rome, Rome, Italy; ^5^Department of Anesthesiology and Intensive Care, Catholic University of Sacred Heart Rome, Rome, Italy; ^6^Software House INTECS S.p.A., Rome, Italy; ^7^Department of Obstetrical-Gynecological Sciences and Urologic Sciences, Sapienza University of Rome, Rome, Italy

**Keywords:** older adults, chronic kidney disease, cardiovascular disease, inflammation, weight loss

## Abstract

**Background:**

Chronic kidney disease is a common condition in the general population, particularly among older adults. Renal impairment is in turn associated with metabolic and nutritional derangements and with increased risk of cardiovascular disease.

**Aim:**

To compare the metabolic, nutritional, and cardiovascular impact of reduced kidney function between patients with and without known renal disease.

**Materials and Methods:**

We enrolled consecutive outpatients (age ≥65 years) with reduced renal function who were divided into two groups: Group A with history of renal disease and Group B with unknown renal disease. Metabolic and nutritional parameters, including involuntary body weight loss (BWL) in the previous 6 months, mineral metabolism, inflammatory indices, and left ventricular mass index (LVMI), were evaluated.

**Results:**

A total of 76 patients were enrolled. Group A (*n* = 39, M: 24, F: 15) showed greater BWL with a significant reduction of 25-hydroxyvitamin D, transferrin, cholinesterase, albumin, and LVMI with respect to Group B (*p* < 0.01). Conversely, Group B (*n* = 37, M: 23, F: 14) showed significantly increased intact parathyroid hormone, total cholesterol, low-density lipoprotein, triglycerides, and C-reactive protein when compared to Group A (*p* < 0.05).

**Conclusion:**

The positive history of renal disease may negatively impact on several metabolic and nutritional parameters related to increased cardiovascular risk among older adults.

## Introduction

Chronic kidney disease (CKD) is a common condition and it is increasing worldwide, particularly in adults aged ≥70 years, where the prevalence in the United States, Europe, and China is 47, 35, and 28%, respectively (1, 2). The current Kidney Disease Outcomes Quality Initiative (KDOQI) classification system for CKD is mainly based on the reduction of estimated glomerular filtration rate (eGFR), which should be persistent for more than 3 months, and it is classified in five stages (3). Epidemiological studies using this classification have highlighted that approximately 10% of the adults and 20–54% of the older adults suffer from CKD in stages 3–5 (4, 5). However, we do not have yet a nomogram of the measurement in a large sample of healthy individuals across the age spectrum of 20–80 years (5–7). The prevalence of CKD is particularly high in older adults, although the risk for progression to end-stage renal disease (ESRD) among older CKD patients is low. Therefore, the diagnosis of CKD should require an eGFR inappropriately low, as a consequence of a known disease, and/or the presence of abnormally elevated proteinuria or albuminuria (8–10). In particular, Hallan et al. (9) suggested to consider in CKD stage 3 the presence of micro-, normo-, or macro-albuminuria to improve the diagnostic accuracy for the progression to ESRD. Several studies, including the Nord-Trøndelag Health Study 2 (11–13), showed that lower eGFR and increased albuminuria in 24-h urine collection and increased albumin:creatinine ratio are relevant independent risk factors among older adults.

The question then arises whether this lower eGFR is a consequence of known kidney disease or if it is the result of aging and whether a stable low eGFR in older adults is sufficient or not to satisfy a homeostatic demand. Homeostatic failure occurs when eGFR is <60 ml/min/1.73 m^2^ and, with higher prevalence, when it is <45 ml/min/1.73 m^2^ (1, 13).

The aim of this study was to compare patients with known renal disease and those with eGFR <60 ml/min without known disease and to evaluate whether this differentially impacts patients’ metabolic, nutritional, and cardiovascular parameters.

## Materials and Methods

### Study Design and Subjects

We performed an observational study on consecutive stable outpatients from the Nephrology Unit of Policlinico Umberto I, Sapienza University of Rome, Italy. The study was approved by the clinical research ethics committee of our University Hospital. The study conforms to the principles outlined in the Declaration of Helsinki and we obtained a written consent from each patient enrolled in this study. Patients with eGFR 30–60 ml/min were recruited in the period from January 2013 to August 2015. Exclusion criteria were heart failure, underlying malignancy, chronic liver disease, chronic obstructive pulmonary disease, cerebrovascular disease, acute myocardial infarction, and acute coronary syndrome reported in the previous 3 months. We also excluded patients who refused to give consent and patients with missing data. Patients were divided into two groups: Group A included patients with known renal disease and Group B those with eGFR <60 ml/min without known renal disease.

The Chronic Kidney Disease Epidemiology Collaboration equation was used to calculate eGFR (5, 14). Additionally, the presence of comorbidities, including diabetes and arterial hypertension, and current therapies were collected. Patients in both groups were allowed to continue their therapy with statins, antihypertensive, and antiplatelet drugs.

### Anthropometric Assessment

Body weight was determined to the nearest 0.1 kg using a calibrated digital scale. Body mass index (BMI) was calculated by the formula [weight (kg)/height^2^ (m^2^)]. We also calculated the body weight loss in the previous 6 months.

### Laboratory Measurements

Blood samples were collected after an overnight fasting of at least 12 h. In all patients, the levels of fasting plasma glucose, total serum cholesterol, high-density lipoprotein (HDL), low-density lipoprotein (LDL), triglycerides, Blood Urea Nitrogen (BUN), serum calcium, phosphate, sodium, potassium, serum uric acid, iron metabolism, including transferrin, cholinesterase, C-reactive protein (CRP), and erythrocyte sedimentation rate (ESR) were measured using standard automated techniques. Serum albumin was determined by bromocresol purple method. Parathyroid hormone was measured using a two-site assay that measures “intact” hormone (iPTH) (pg/mL) and 25-hydroxyvitamin D (25-OH Vit D) (ng/mL) by radioimmunoassay. LDL cholesterol was calculated using the Friedewald equation: LDL = total cholesterol − HDL − (triglycerides/5). Arterial blood gas analysis was performed using a blood gas analyzer (Nova Phox Plus C). We have measured albuminuria in 24-h urine (15).

### Echocardiography

M-mode 2D echocardiographic examinations by a single experienced sonographer in the echocardiography laboratory and using a standard institutional protocol were completed. Commercially available instruments (Toshiba Aplio xV, Toshiba American Medical Systems, Inc., Tustin, CA, USA) equipped with 2.25- to 7.5-MHz imaging transducers were used. The subjects were in the left decubitus position, and the sonographer was blinded to all clinical details of the patients (16). All echocardiographic data according to the guidelines of the American Society of Echocardiography were recorded (17). The end-diastolic and end-systolic left ventricular internal diameter (LVED and LVES, respectively), interventricular septum thickness, and posterior wall thickness were measured. The left ventricular mass by Devereux’s formula normalized by body surface area and height was estimated, and its index (LVMI) was also calculated (18).

### Statistical Analysis

Data management and analysis were performed using the IBM^®^ SPSS^®^ Statistics 18 for Windows^®^ software (IBM Corporation, Armonk, NY, USA). The normality of variables was tested by the method of Kolmogorov–Smirnov test for normal distributions. All continuous variables were expressed as mean ± SD, and categorical variables were expressed as number (percentage). Student’s *t*-test or Mann–Whitney *U*-test was performed to determine differences between groups. Binomial test or Chi-square test was used for comparison of categorical data. Pearson’s or Spearman’s correlation was used to determine the relationship and the strength of association between the variables in bivariate correlation. Potential confounders, such as age and gender, were included in the analyses when comparing the two groups. A *p* value < 0.05 was considered statistically significant.

## Results

The study included 76 consecutive patients, 29 females and 47 males, with a mean age of 70 ± 5.4 years.

Patient’s characteristics are shown in Table 1. There were no significant differences between the two groups regarding sex, age, eGFR (mL/min), creatinine (mg/dL), sodium (mEq/L), potassium (mEq/L), calcium (mg/dL), phosphorus (mg/dL), serum uric acid (mg/dL), HDL (mg/dL), and bicarbonates (mEq/L) (Tables 1 and 2).

**Table 1:** **Patient’s demographic and clinical characteristics**.

*N* patients = 76	Group A (*N* = 39)	Group B (*N* = 37)	*p*
Female (*N*)	15	14	0.86
Age (year)	71.2 ± 4.4	72.3 ± 4.3	0.27
Body mass index (kg/m^2^)	23.5 ± 3.8	26.1 ± 3.5	**0.003**
eGFR (mL/min)	48.2 ± 10.3	52.6 ± 10.5	0.07
BUN (mg/dL)	48.13 ± 16.49	31.49 ± 8.73	**<0.001**
Creatinine (mg/dL)	1.7 ± 0.42	1.6 ± 0.54	0.37
Sodium (mEq/L)	139.1 ± 2.1	139.3 ± 1.8	0.66
Potassium (mEq/L)	4.1 ± 0.7	4.0 ± 0.5	0.48
Calcium (mg/dL)	9.2 ± 0.3	9.4 ± 0.6	0.07
Phosphorus (mg/dL)	4.5 ± 0.5	4.3 ± 0.4	0.06
Arterial hypertension (*N*)	38	15	**<0.0001**
Diabetes mellitus (*N*)	27	0	**<0.0001**
Chronic pyelonephritis (*N*)	3	0	0.26
Chronic glomerulonephritis (*N*)	7	0	**0.02**

*Data are shown as mean ± SD*.

*eGFR, estimated glomerular filtration rate; BUN, Blood Urea Nitrogen*.

*Bold values indicate statistically significant p values*.

**Table 2:** **Patient’s nutritional, metabolic, inflammatory, and cardiac variables**.

*N* patients = 76	Group A (*N* = 39)	Group B (*N* = 37)	*p*
Albumin (g/dL)	3.73 ± 0.51	4.69 ± 0.40	**<0.0001**
Cholinesterase (UI/mL)	7312 ± 2864	9647 ± 3637	**0.003**
Transferrin (mg/dL)	218.40 ± 37.68	266.22 ± 78.71	**0.001**
Hemoglobin (g/dL)	12.0 ± 0.9	14.2 ± 5.6	**0.015**
25-OHVitD (ng/mL)	15.36 ± 10.65	26.06 ± 12.91	**<0.0001**
iPTH (pg/mL)	91.4 ± 57.7	67.6 ± 29.9	**0.03**
Total cholesterol (mg/dL)	190 ± 32	163 ± 25	**<0.001**
HDL (mg/dL)	50.2 ± 11.8	44.8 ± 12.0	0.05
LDL (mg/dL)	112.0 ± 37.1	95.9 ± 21.0	**0.02**
Triglycerides (mg/dL)	155 ± 71	118 ± 47	**0.01**
Serum uric acid (mg/dL)	4.7 ± 1.1	4.3 ± 0.9	0.09
pH	7.35 ± 0.04	7.40 ± 0.03	**<0.0001**
Bicarbonates (mEq/L)	21.7 ± 4.1	23.0 ± 1.8	0.10
CRP^a^ (mg/dL)	0.23 (0.13; 0.31)	0.13 (0.08; 0.29)	**0.046**
ESR (mm/h)	27.7 ± 16.9	16.3 ± 11.2	**<0.0001**
Body weight loss^a^ (%)	3.19 (0; 5.97)	1.78 (−4.05; 1.83)	**0.009**
LVMI (g/m^2^)	141 ± 16	103 ± 20	**<0.0001**

*Data are shown as mean ± SD*.

*^a^Median (interquartile range) is shown for non-normally distributed variable*.

*25-OHVitD, 25-hydroxyvitamin D; iPTH, intact parathyroid hormone; HDL, high-density lipoprotein; LDL, low-density lipoprotein; CRP, C-reactive protein; ESR, erythrocyte sedimentation rate; LVMI, left ventricular mass index*.

*Bold values indicate statistically significant p values*.

Group A showed lower albumin (g/dL) (*p* < 0.0001), cholinesterase (UI/mL) (*p* = 0.003), transferrin (mg/dL) (*p* = 0.001), hemoglobin (g/dL) (*p* = 0.015), 25-OHVitD (ng/mL) (*p* < 0.001), and pH (*p* < 0.0001) with respect to Group B (Table 2). Moreover, Group A showed greater body weight loss (BWL) in the previous 6 months with respect to Group B (*p* = 0.009), higher LVMI g/m^2^ (*p* < 0.0001), BUN (mg/dL) (*p* < 0.001), iPTH (pg/mL) (*p* = 0.03), total cholesterol (mg/dL) (*p* = 0.02), LDL (mg/dL) (*p* = 0.04), triglycerides (mg/dL) (*p* = 0.02), CRP (mg/dL) (*p* = 0.046), and ESR (mm/h) (*p* = <0.0001) (Tables 1 and 2). The patients in Group B had reached the target blood pressure level by monotherapy, while those in Group A were in treatment with polytherapy. Proteinuria showed a different behavior in Group A and B. In fact, in Group A 16 out of 39 patients (41%) had proteinuria >1 g/24 h. In Group B, proteinuria was present in only 7 out of 37 patients (19%), and it was <1 g/24 h.

## Discussion

The results obtained in the present study would suggest that age-related compared to renal disease-related decline in renal function differentially impacts on patients’ metabolic and nutritional parameters, and possibly on cardiovascular disease (CVD) risk.

Chronic kidney disease is a common feature among older adults and less frequently progresses to ESRD with respect to younger patients (13). CVD is the most common cause of morbidity and mortality associated with CKD, due to well-known cardiovascular risk factors in older patients and to several novel risk factors associated with CKD, such as abnormal nutritional and metabolic parameters.

In a large number of CKD patients most of these risk factors are present when eGFR is 60 ml/min/1.73 m^2^ or less (13). However, the relative risk associated with traditional risk factors, such as hypertension and hypercholesterolemia, is lower in older patients compared to younger ones. In older patients, there is an inverse relationship between traditional CVD risk factors, such as BMI, blood pressure, cholesterol, and all-cause mortality (19), while altered nutritional and metabolic derangements may represent negative prognostic factors in CKD.

In our study, we found that older adults with known renal disease have a worse nutritional and metabolic status with respect to patients without a known renal disease. This is reflected by the presence of alteration of metabolic and inflammatory profile and by altered cardiovascular index (LVMI), although the eGFR was comparable between the two groups.

Only patients in Group B achieved the lipid target range recommended by the European and Canadian guidelines (20), although the recent American guidelines give more importance to the modification of lifestyles, instead of targeting strict lipid levels (21). Considering the high prevalence of diabetes among patients in Group A, the cholesterol levels were above the recommended range, representing a further relevant risk factor for cardiovascular events. Also, triglycerides were significantly higher in Group A with respect to Group B (>150 mg/dl) (20).

The KDOQI classification considers the eGFR of 50 ml/min/1.73 m^2^ as indicative of moderate CKD (stage 3), independent of age. It has been recognized that the physiological aging process of the kidney leads to a loss of GFR at a rate of about 1 ml/min/1.73 m^2^ per year after 40 years of age (22, 23). Two large studies found that moderate reductions in eGFR were not associated with increased mortality among older adults (i.e., ≥60 years of age in one study and ≥65 years in the other study) (24, 25). In contrast, in a meta-analysis performed by the Chronic Kidney Disease Prognosis Consortium (26), an association was present between eGFR <60 ml/min/1.73 m^2^ and increased all-cause and cardiovascular mortality among adults ≤65 years of age and their counterparts ≥65 years of age (27).

Conversely, in a large cohort of veterans who fulfilled criteria for CKD stage 3 or higher, the prognostic implications of eGFR for death and ESRD varied greatly depending on the age (28). Moreover, Davies and Shock (29) showed that mean GFR (measured using the gold standard of inulin clearance) decreased from 123 ml/min/1.73 m^2^ at age 20–29 years to 65 ml/min/1.73 m^2^ at age 80–89 years.

Cross-sectional evaluations of measured GFR and eGFR in healthy individuals, e.g., potential kidney donors, showed a reduction in the mean value of 6–8 ml/min/1.73 m^2^ per decade (30). This indicates that there is a real reduction in renal function among older adults. Available data showed that, at least until the age of 80 years, the renal functional reserve, i.e., the increase in eGFR after an amino acid load, was preserved in these subjects independent of gender (31, 32). Hollenberg et al. (33) demonstrated a reduction of the renal vasodilatory response to acetylcholine in older kidney donors. Furthermore, the arterial wall is substantially remodeled with aging even in the healthy subjects. Histological changes such as glomerulosclerosis, tubular atrophy, interstitial fibrosis, and arteriolar sclerosis, which are typical findings of the common pathway of progressive CKD, are increasingly found with aging even in the absence of hypertension and diabetes or other chronic diseases (4, 12). Data from the PREVEND study have shown that eGFR were independently and significantly associated with cardiovascular events only in subjects <60 years of age (34). Indeed, a stable low eGFR in older adults, provided that it is physiologically sufficient to meet homeostatic demands, cannot be considered as a disease *per se* and rarely progresses to kidney failure, even if it implies a reduced reserve and vulnerability to drugs overdosing and increased risk of acute kidney injury (AKI) (13).

The complications related to reduced eGFR would be abnormal sodium balance causing hypertension, a blunted erythropoietin axis causing anemia, and disturbed calcium and phosphate balance causing secondary hyperparathyroidism and metabolic acidosis (11).

In our study, the homeostatic and metabolic disorders were significantly increased in patients with positive history of renal disease (Group A), although also patients with no history for disease (Group B) showed depletion of vitamin D. In particular, Group A showed a more pronounced depletion of vitamin D with respect to Group B, being associated with a small but significant increase of iPTH. Moreover, Group A showed a worsening trend of nutritional status, with a significant reduction in albumin and cholinesterase levels, lower BMI, and greater BWL in the previous 6 months. In Group A, we also found a significant reduction in hemoglobin levels, although these values were within the therapeutic range for patients with eGFR ≤60 ml/min (35), and they were associated with mild iron deficiency and increased inflammatory status. We observed significant increase of LVMI in Group A, which might be influenced by several factors, including hyperparathyroidism, and by the higher rate of hypertension documented within this group. Based on these observations, patients with history of renal disease may require a more careful follow-up visit, with assessment of nutritional and metabolic status compared with those with similar eGFR but with unknown underlying renal disease. These findings question the clinical relevance of a standard stage-based approach to the management of CKD, which is supported in most of the available international guidelines and focus on the critical need to use stronger prognostic tools for an early identification of older adults who will progress from CKD to ESRD, CVD, or AKI (36). Moreover, the current staging system for CKD is largely based on the concept that GFR ≥90 ml/min/1.73 m^2^ is normal, 60–89 ml/min/1.73 m^2^ is reduced, and ≤60 ml/min/1.73 m^2^ is always abnormal, independent of the presence of kidney damage (37, 38). This approach does not take into account the biological variability and the renal reserve (39, 40).

The average eGFR is variably decreased in older adults, as a consequence of biological aging and underlying comorbidities (41). In some patients, the decline of renal function is enhanced by concomitant CVDs, and a low eGFR may be the consequence of aging and concomitant underlying diseases (42, 43).

As supported by other evidences, in older patients with CKD we should consider not only the eGFR and the presence of proteinuria but also the underlying renal disease (44, 45). Moreover, we should assess in this population the nutritional and metabolic status in order to better stratify cardiovascular risk and to implement tailored therapeutic interventions (46, 47).

We acknowledge the limitations of our study. The small sample size might have limited the possibility to recognize associations between patients’ clinical characteristics. In this light, the present cohort of patients may not be representative of the entire CKD outpatient population. In particular, we included a large proportion of patients on antiplatelet and antihypertensive therapies and on statins and the potential impact of hypertension, hypercholesterolemia, and their treatments on LVMI may have potentially confounded our results. Although both groups were affected by hypertension, Group A had a higher prevalence of this condition probably determining the significant difference observed in LVMI. Approximately 40% of patients in Group B suffered hypertension. This may be associated with the reduction of eGFR. However, in contrast with Group A, patients in Group B were treated with antihypertensive monotherapy, showing blood pressure values within normal range.

## Conclusion

In spite of comparable eGFR, older adults with underlying renal disease have a worse nutritional and metabolic balance with respect to patients with negative history. This is reflected by the presence of alterations of metabolic and inflammatory profile and by impaired cardiovascular index.

Integrated care for older adults with loss of renal function should go beyond a focus on reduced eGFR or proteinuria and include consideration of underlying cause of kidney disease.

Based on our results, it might be helpful to prospectively evaluate the impact of nutritional alterations on clinical outcomes and health-care costs in older adults with impaired renal function.

## Author Contributions

SL, MIA, MM, and AM made substantial contributions to conception and design and interpretation of data; SA, DM, and AD made substantial contributions to acquisition of data; and CL, PA, and MC made substantial contributions to analysis of data. All authors participated in drafting the article or revising it critically for important intellectual content. All authors gave final approval of the version to be submitted.

## Disclaimer

The authors are responsible for the content and writing of the paper. The manuscript is not under consideration for publication elsewhere.

## Conflict of Interest Statement

The authors declare that the research was conducted in the absence of any commercial or financial relationships that could be construed as a potential conflict of interest. The reviewers CO and AD and handling Editor declared their shared affiliation and the handling Editor states that the process nevertheless met the standards of a fair and objective review.
