# Corrigendum: Effect of Underlying Renal Disease on Nutritional and Metabolic Profile of Older Adults with Reduced Renal Function

**DOI:** 10.3389/fnut.2017.00044

**Published:** 2017-10-04

**Authors:** Silvia Lai, Maria Ida Amabile, Silvia Altieri, Daniela Mastroluca, Carlo Lai, Paola Aceto, Massimiliano Crudo, Anna Rita D’Angelo, Maurizio Muscaritoli, Alessio Molfino

**Affiliations:** ^1^Department of Clinical Medicine, Sapienza University of Rome, Rome, Italy; ^2^Department of Clinical and Molecular Medicine, Sapienza University of Rome, UOC Nephrology, Sant’Andrea Hospital, Rome, Italy; ^3^Nephrology and Dialysis Unit, Hospital ICOT Latina, Sapienza University of Rome, Rome, Italy; ^4^Department of Dynamic and Clinic Psychology, Sapienza University of Rome, Rome, Italy; ^5^Department of Anesthesiology and Intensive Care, Catholic University of Sacred Heart Rome, Rome, Italy; ^6^Software House INTECS S.p.A., Rome, Italy; ^7^Department of Obstetrical-Gynecological Sciences and Urologic Sciences, Sapienza University of Rome, Rome, Italy

**Keywords:** older adults, chronic kidney disease, cardiovascular disease, inflammation, weight loss

In the original article, there was an error, in particular, in the results section of the Abstract:

**Results:** A total of 76 patients were enrolled. Group A (*n* = 39, M: 24, F: 15) showed greater BWL with a significant reduction of 25-hydroxyvitamin D, transferrin, cholinesterase, albumin, and LVMI with respect to Group B (*p* < 0.01). Conversely, Group B (*n* = 37, M: 23, F: 14) showed significantly increased intact parathyroid hormone, total cholesterol, low-density lipoprotein, triglycerides, and C-reactive protein when compared to Group A (*p* < 0.05).

A correction has been made in the results section of the Abstract.

**Results:** A total of 76 patients were enrolled. Group A (*n* = 39, M: 24, F: 15) showed greater BWL with a significant reduction of 25-hydroxyvitamin D, transferrin, cholinesterase, albumin, and greater LVMI with respect to Group B (*n* = 37, M: 23, F: 14) (*p* < 0.01). In addition, Group A showed significantly increased intact parathyroid hormone, total cholesterol, low-density lipoprotein, triglycerides, and C-reactive protein when compared to Group B (*p* < 0.05).

The authors declare that this error, present only in the abstract, does not change the scientific conclusions of the article in any way.

## Conflict of Interest Statement

The authors declare that the research was conducted in the absence of any commercial or financial relationships that could be construed as a potential conflict of interest. The reviewer MD and handling editor declared their shared affiliation.

